# Sleeve material, height, and drill surface changes in guided endodontics: Vicker’s hardness and scanning electron microscope evaluation (A pilot study)

**DOI:** 10.1186/s12903-025-07560-4

**Published:** 2026-01-05

**Authors:** Anna Muryani, Dudi Aripin, Hendra Dian Adhita Dharsono, Satrio Wicaksono, Zainul Ahmad Rajion, Brigita Nadia Wirawan, Aloysius Kiyoshi Sumo Wardoyo, Wandi Prasetia

**Affiliations:** 1 Doctoral Programme Faculty of Medicine, Universitas Padjadjaran, Bandung, West Java Indonesia; 2 Conservative Dentistry Department Faculty of Dentistry, Universitas Padjadjaran, Bandung, West Java Indonesia; 3 Faculty of Mechanical and Aerospace Engineering, Institut Teknologi Bandung, Bandung, West Java Indonesia; 4 Department Oral Maxillofacial Surgery and Oral Diagnosis Kulliyyah of Dentistry, International Islamic University Malaysia, Kuantan, Malaysia

**Keywords:** Guided endodontics, Endodontic guide sleeve, Vickers hardness, Drill wear, Scanning electron microscopy, Sleeve material, Sleeve height

## Abstract

**Background:**

Guided endodontic access uses a CBCT-derived template with a guide sleeve to direct the drill for precise canal location, particularly in calcified teeth. This technique is more conservative, time-efficient, and accurate than conventional methods. Guide sleeves are fabricated from various materials (e.g., zirconia ceramic, cobalt-chromium alloy, titanium alloy, or 3D-printed resin), but their hardness and durability under drilling stress are not well characterized. However, guiding long, narrow drills through sleeves may generate excess heat due to friction between the surfaces, potentially causing tool wear. Studies in implant dentistry show significant drill wear with repeated use, but the effects of sleeve material and sleeve height on endodontic drill wear remain uncharacterized. This study used Scanning Electron Microscopy (SEM) to examine drill surface changes after guided Minimally Invasive Endodontic Access (MIEA) with different sleeve materials (resin, zirconia [Y-TZP], Ti-6Al-4 V, CoCr alloy) and heights. This study evaluated Sleeve Material, Height, and Drill Surface Changes in Guided Endodontics through the Vickers Hardness Test and Scanning Electron Microscope.

**Methods:**

In vitro, 30 guide sleeves (10 sizes: 3 mm, 5 mm, 7 mm; 4 different materials) fit a 1.5 mm carbide bur with limited clearance. Sleeve materials tested in the current study are dental photopolymer resin, Y-TZP, Co–Cr, and Ti-6Al-4 V. Before and after drilling, the sleeve hardness was measured through Vickers Hardness Test (HV0.2). Hardness variations (ΔVHN) were determined. A one-way ANOVA was used to assess hardness among materials and sleeve heights, with significance at *p* < 0.05. Additionally, three teeth were drilled under irrigation by one group of standardized high-speed carbide drills per guide. Cleaning and imaging of drill tips and flutes were done using a SEM (1000x magnification). Two examiners rated wear as Grade 0 (no wear), Grade 1 (minor blunting or edge rounding), or Grade 2 (severe chipping or edge loss). Five drills showed a noticeable thermal discoloration (“burn marks”) and were included in the analysis. This study is a pilot study or an early feasibility study. The data will be analysed using a Descriptive approach.

**Results:**

While zirconia sleeves had the highest hardness rating (mean ~ 1392 HV pre-drilling; ~1389 HV post-drilling), resin sleeves had the lowest (~ 6 to 7 HV both pre- and post-drilling), while CoCr and Ti-alloy sleeves had intermediate hardness (~ 315 and ~ 405 HV). ANOVA showed significant differences in material type (*p* < 0.001), but no significant effect of sleeve height on hardness (*p* > 0.8). Due to modest hardness variations, drilling cycles do not strain-harden or soften the sleeve. Four drills (30%) exhibited Grade 1 wear. Six drills (70%) had Grade 2 wear. Medium- and short-resin sleeves showed Grade 1 wear, whereas Zirconia, Titanium, and CoCr showed Grade 2 wear. Short sleeves produced Grade 1 wear, while medium/long sleeves frequently caused Grade 2 wear. Five drills with burn marks were classified as Grade 2. Overall, Grade 2 wear was observed in 60% of drills, and Grade 1 in 40%.

**Conclusions:**

Sleeve hardness was primarily determined by the material type, with sleeve height having no significant effect. Zirconia guide sleeves were harder than CoCr, Ti, and resin, and may also offer greater wear resistance and durability for guided endodontic template sleeves. All tested materials maintained their hardness after simulated guided access drilling. Guided minimally invasive endodontic access drill wear was considerable with static sleeves. The majority of the drills experienced thermal damage and were classified as Grade 2 surface damage. Drill abrasion was notably increased when using resin, zirconia, and cobalt-chromium (CoCr) sleeves, attributed to their greater hardness, wear resistance, and sleeve height. While resin sleeves may help reduce wear during minimally invasive guided endodontics, the absence of an effective root canal irrigation system limits cooling efficiency. Clinicians must be aware that more rigid guides and sleeve systems may demand additional measures, such as optimized irrigation or intermittent drilling, to avoid thermal and mechanical damage. Proper selection of guide sleeves and implementation of effective cooling mechanisms are essential to the success of guided endodontic procedures.

## Introduction

Guided endodontics uses CBCT imaging and computerized planning to generate a resin template with a cylindrical bur guide. This method provides precise access to calcified or occluded canals, preserving tooth structure and the operator [1–4]. The sleeve material keeps the bur on track in static guiding. Guided endodontics may use high-strength ceramics, such as Zirconia, metals, or 3D-polymer resins for sleeves [5–8]. Additionally, sleeve interiors are commonly coated or composed of hard material (e.g., ≥ 1800 HV coatings) to prevent wear and friction during drilling [5–7, 9, 10]. 

This method offers high accuracy and requires operator expertise [11]. However, lengthy, narrow, high-speed drills through inflexible sleeves pose additional hazards. Heat and abrasion may occur due to friction between the sleeve and the drill bit. Implant osteotomy studies demonstrated that drills undergo wear and heat generation after repeated use [12]. The geometry of guided endodontic drills is smaller (< 1.5 mm) and longer than implant drills, resulting in reduced bulk and increased heat generation [13]. To support the drill, sleeves should be ≥ 5 mm in height. However, higher sleeves might hinder coolant flow and irrigation, leading to overheating [14]. Different sleeve materials (soft resin vs. hard metal/ceramic) affect friction between surfaces and surface wear. Despite these concerns, there are still limited studies related to how sleeve material and length affect the wear of endodontic drill [12, 14]. 

This study examines how variations in sleeve material and height affect the hardness and wear characteristics of guided endodontic drills. Resin, zirconia, titanium alloy, and CoCr sleeves at heights of 3, 5, and 7 mm were investigated for changes in hardness and the resulting drill surface changes. Recent dental provider data and clinically relevant research offer endodontists new insights into the relationship between sleeve materials and drill performance. Sleeve selection significantly influences hardness stability and surface integrity. Although zirconia offers superior hardness, stability, and drill surface integrity, it can increase drill wear and heat generation under poor irrigation. These findings emphasize the importance of balancing access accuracy, sleeve durability, and thermal safety in clinical practice. Despite these advances, nothing is known about how guided sleeve material impacts sleeve performance. Friction and heat generated during drilling might work-harden or soften the sleeve. Higher-hardness materials may deflect the drill differently from lower-hardness materials. For instance, Connert et al. found that dental material hardness affects drill trajectories, indicating that sleeve hardness may also be necessary. Furthermore, sleeve deformation or wear may reduce guide accuracy with repeated applications, underscoring the therapeutic significance of sleeve hardness [13]. 

The Vickers hardness of dental photopolymer resin (Resin), zirconia ceramic (Zr), titanium alloy (Ti), and cobalt-chromium alloy (CoCr) sleeves of three varying heights was in vitro tested prior to and following a standard guided endodontic access method. Additionally, the present study investigated whether drilling toughness was influenced by sleeve height (3, 5, or 7 mm above the template). Additionally, the present study investigated the extent of drill surface injury that occurred during guided MIEA in extracted teeth and employed SEM evaluation to address this lacuna. The present study examined four sleeve material types at three different heights. Drill wear was subjectively assessed as Grade 0, Grade 1, and Grade 2 in accordance with previous SEM studies. The null hypothesis is that sleeve hardness and drill attrition during guided endodontic access are not significantly influenced by sleeve material or sleeve height. This study aims to evaluate the influence of sleeve material and height on guided endodontic drill wear and hardness through Vickers microhardness and SEM assessments.

## Methods

Ethical approval for the use of extracted human teeth in this endodontic and Static Guided Endodontic Access (SGEA) investigation was granted by the Institutional Review Board of Research Ethics Committee of Padjadjaran University (Nomor: 194/UN6.KEP/EC/2025). All teeth were extracted for therapeutic purposes based on clinical indications, and written informed consent was obtained from all patients for their use in research. This study was designed as pilot research to evaluate the effect of sleeve material and sleeve height on guided endodontic drill wear and hardness. Eight experimental groups were established according to variations in sleeve material and height. The sample size was determined through an a priori power analysis using G*Power software (version 3.1; Heinrich-Heine-Universität Düsseldorf, Germany) [15]. The parameters applied included a large effect size (Cohen’s f = 0.40), a significance level of = 0.10, and a statistical power of 0.90, which indicated a minimum requirement of three specimens per group, yielding a total of 30 samples. As this was a pilot study, the sample size was considered adequate to generate preliminary data and to inform future confirmatory investigations. The sample used for the drill evaluation was descriptive, intended to characterise wear and hardness patterns rather than to conduct inferential statistical comparisons.

### Sample preparation

The present in vitro study assessed sleeve-guided endodontic hardness with various materials at different heights before and after guided MIEA. Using all possible combinations of the four sleeve materials and the three sleeve heights, thirty custom endodontic guide sleeves were made (*n* = 30; 3 samples per group). The following materials were assessed for the sleeves: (1) yttria-stabilized zirconia (Y-TZP ceramic), (2) chrome-cobalt alloy (CoCr dental alloy), (3) titanium alloy (medical-grade Ti-6Al-4 V, which served as the traditional control), and (4) a dental photopolymer resin that was 3D printed. Each sleeve was a cylindrical insert designed to fit a standard guide template and provide minimal clearance for the guiding bur. The study randomly allocated specimens into sleeve-guided endodontic categories: Zr 3 mm, Zr 5 mm, Zr 7 mm, CoCr 3 mm, CoCr 5 mm, CoCr 7 mm, Ti 5 mm, Resin 3 mm, Resin 5 mm, and Resin 7 mm, prior to and during minimally invasive endodontic access cavity preparation to assess micro-hardness. The inner diameter of each sleeve corresponded to the diameter of the long-shank carbide bur (about 1.5 mm shaft) used for access preparation, enabling minimal clearance (approximately 0.2 mm) for unobstructed rotation. The outside diameter of each sleeve was engineered to fit securely inside the tube housing of the guide.

Three different sleeve heights (3 mm, 5 mm, and 7 mm) were manufactured for each material. Height measurements were taken from the upper surface of the template to the upper extremity of the sleeve. All sleeves were manufactured to precise tolerances: resin sleeves were produced using a high-resolution stereolithography printer (layer thickness 25 μm), zirconia sleeves were machined from pre-sintered blocks and sintered, and metal sleeves (Ti and CoCr) were milled. Prior to testing, each sleeve’s inner surface was ultrasonically cleaned, polished as required, and its baseline hardness was measured.

This study used a random sample of 30 teeth, including upper and lower incisors, canines, and premolars. The inclusion criteria included teeth devoid of caries, restorations, root resorption, and fractures; teeth without root canal obliteration; and teeth exhibiting radiographically straight root canals. Exclusion criteria included teeth with more than two roots, open apices, and deciduous teeth, as determined by periapical radiographs.

**Fig. 1:**
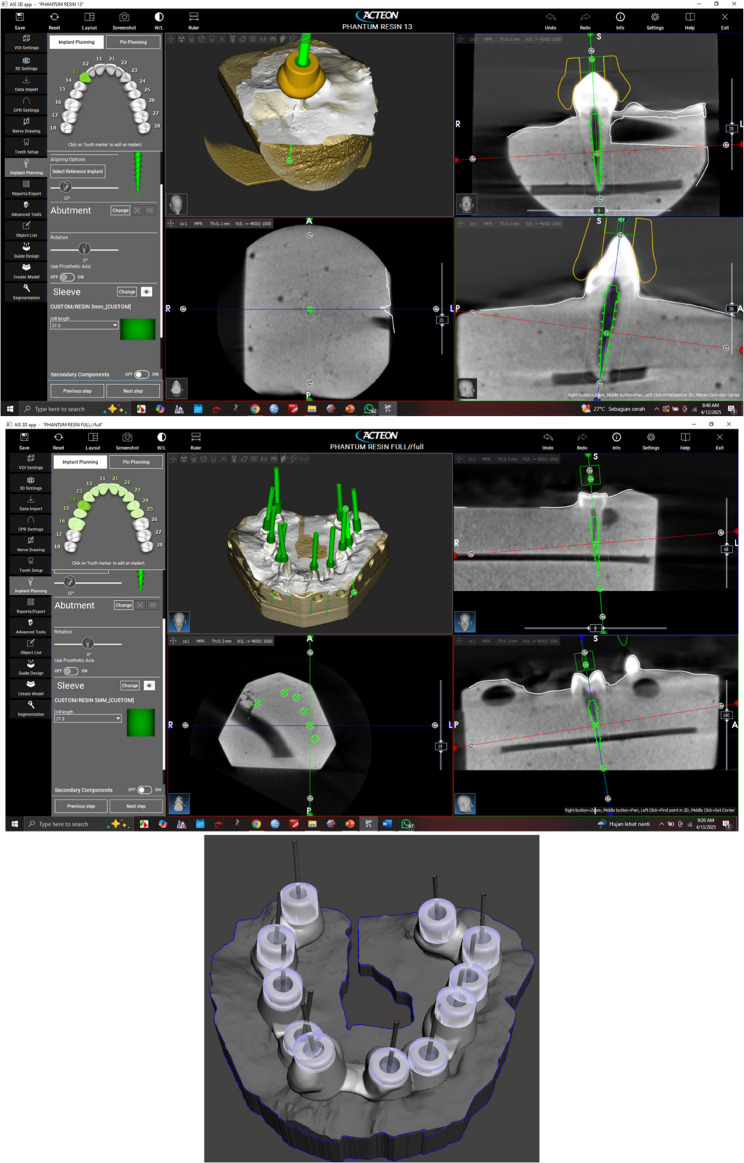
Designing the guided endodontic template

**Fig. 2:**
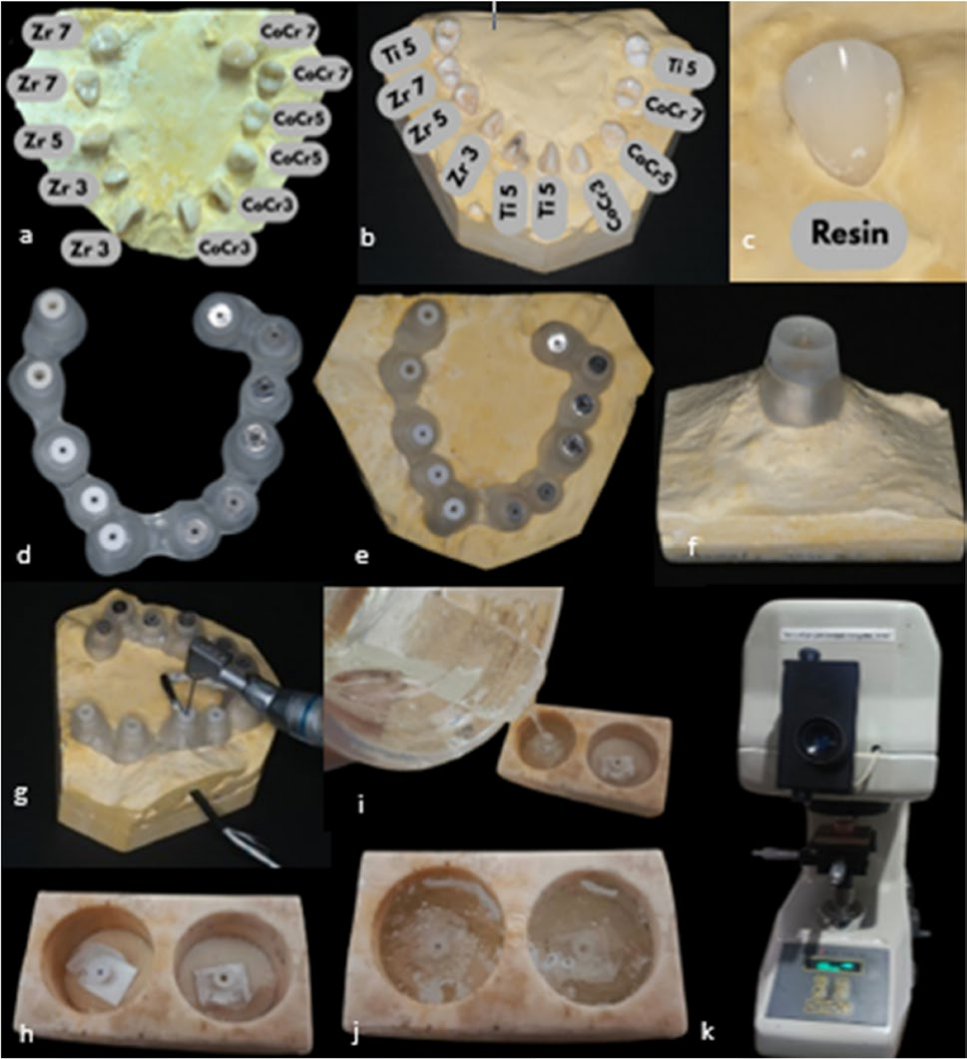
**a**-**f** The guided endodontic access with variations in material and height. Minimally invasive endodontic access cavity preparation, **h**-**j**. Micro Vickers hardness tester samples (cold mounting), **k**. Micro Vickers Hardness Tester Samples (Cold mounting) and Hardness Tester (Zwick/Roell ZHVµ-S, Zwick Roell, Germany)

To create the appearance of an arch, extracted human teeth were collected and placed in type III dental stone. After a cone-beam computed tomography (CBCT) scan of the specimen, planning software (Acteon Imaging Suite 3D, Acteon Group, France) was used to digitally create a guided endodontic access template (Fig. 1). The template had reusable sleeve inserts and was made with biocompatible resin using a 3D printer (Formlabs Form 4B, USA). Each sleeve was required to be inserted into the guiding cylinder of the template for each test, ensuring it was aligned with the designated canal access trajectory (Fig. 2a and F).

## Guided endodontic access protocol

A skilled operator performed all guided access preparations to minimize differences among operators. After placing the guide template on the tooth model, a long-shank carbide endodontic bur with a 1.5 mm diameter cutting tip was used to form the access cavity through the sleeve for each tested sleeve. The bur was installed in a handpiece operating at 1500 RPM. To eliminate debris and prevent the drill from overheating, a “pumping” drilling technique was employed. Additionally, it included pulling the bur back every two to three seconds and moving it forward slowly. To simulate the worst-case situation of insufficient cooling, continuous irrigation was not used during drilling. Drilling continued until the bur’s stopper either hit the sleeve or entered the pulp chamber, indicating the desired depth had been reached. To prevent wear and tear from frequent use, each sleeve was used only once for a single access preparation. The sleeve and template were removed after the access cavity was completed. Then the sleeve was removed from the template for examination. During the procedure, steps were taken to prevent the bur from exerting excessive lateral stress beyond the guide’s limits (Fig. 2g).

## Assessment of Vickers hardness

Samples of endodontic guide sleeves were fabricated from zirconia, chrome cobalt (CoCr), titanium, and resin at heights of 3 mm, 5 mm, and 7 mm. Each sample was cold-mounted in a two-component epoxy resin to ensure stability during testing. After complete polymerization, specimens were sequentially sanded and polished to obtain a smooth, flat surface suitable for Vickers microhardness assessment (Fig. 2h and k).

Vickers microhardness testing was performed according to ISO 6507-1:2018 and ASTM E384-22 standards using a micro-Vickers hardness tester (Zwick/Roell ZHVµ-S, Zwick Roell, Germany) with a 136° diamond pyramidal indenter. A 200 gf (HV0.2) load was applied for 10 s, and indentation diagonals were measured microscopically. The Vickers Hardness Number (VHN) was calculated using HV = 1.854 × F/d², where *F* is the applied load (kgf) and *d* is the mean diagonal length (mm). Three equidistant indentations were made along the inner surface of each sleeve, spaced at least three diagonal lengths apart.

The primary outcome was the change in microhardness of the sleeve’s inner wall before and after guided access drilling. Post-drilling measurements were obtained at corresponding locations, with new indentations made near the original sites to avoid overlap. Differences in pre- and post-drilling VHN values indicated potential surface hardening or softening associated with material deformation, wear, or thermal alteration.

### Data analysis

Data were analysed using SPSS v25 (IBM, USA). Descriptive statistics (mean ± standard deviation) were calculated for the VHN of each material and height group. One-way ANOVA (*p* < 0.005) was used to compare mean VHN values (pre- and post-drilling) among the four material groups, and separately among the three height groups.

## Assessment of SEM

Post-drilling, each used drill was rinsed and dried. The cutting tip and flutes were sputter-coated with gold and examined under a SEM (HITACHI SU3500, Japanese) at 1000× magnification. Both sides and edges of the flutes, as well as the tip, were evaluated using SEM. Two blinded examiners independently scored each drill’s surface wear on a three-point scale adapted from the literature [16]: Grade 0 = no visible alteration; Grade 1 = minor wear or slight blunting of cutting edges; Grade 2 = severe wear (chips or notches) on cutting edges. Discrepancies were resolved by consensus. Drill scoring included five drills that exhibited noticeable thermal discoloration (“burnt marks”); these were recorded but still assigned a 0–2 score. Data were summarized descriptively. The proportion of drills in each wear category was calculated, and wear grades were tabulated by group (Table 1). This study is a pilot study or an early feasibility study. The data will be analysed using a Descriptive approach.

**Table 1:** Overview and comparison of hardness before and after minimal invasive endodontic pulp chamber Preparation by material type and height

			Titanium 5 mm (*n* = 3)	Zirconia 3 mm (*n* = 3)	Zirconia 5 mm (*n* = 3)	Zirconia 7 mm (*n* = 3)	CoCr 3 mm (*n* = 3)	CoCr 5 mm (*n* = 3)	CoCr 7 mm (*n* = 3)	Resin 3 mm (*n* = 3)	Resin 5 mm (*n* = 3)	Resin 7 mm (*n* = 3)
HARDNESS	BEFORE	Mean ± SD	315.6± 2.7	1395.16± 7.1	1330.66± 166.91	1,450.50± 9.01	446.66± 28.96	359.00± 123.00	410.33 ± 45.31	7.66 ± 0.57	6.67 ± 0.57	5.66 ± 2.08
		Median (Min-Max)	317.00 (312.50–317.50)	1,393.50 (1,389.00–1,403.00)	1,422.50 (1,138.00–1,431.50)	1,453.00 (1,440.50–1,458.00)	459.50 (413.50– 467.00)	358 (236.50–482.50)	407.50 (366.50–457.00)	8.00 (7.00–8.00)	7.00 (6.00–7.00)	5.00 (4.00–8.00)
		*p-value*	< 0.001									
	AFTER	Mean ± SD	314.55 ± 4.29	1396.33 ± 6.66	1323.33 ± 171.21	1,447.22 ± 6.67	453.77 ± 28.72	360.44 ± 121.17	408.22 ± 42.51	6.67 ± 0.57	6.33 ± 0.57	5.33 ± 1.52
		Median (Min-Max)	313.33 (311.00- 319.33)	1,394.66 (1,390.67– 1403.67)	1419.00 (1,125.67–1,425.33)	1447.00 (1440.67–1454.00)	468.66 (420.67- 472.00)	368.00 (235.67–477.67.67.67)	407.00 (366.33- 451.33)	7 (6.00–7.00)	6.00 (6.00–7.00)	5.00 (4:00–7:00)
		*p-value*	< 0.001									

*Analysis of the mean difference of normally distributed numerical data was performed using a *One-Way ANOVA* test with significance at *p* < 0.05

## Results

**Fig. 3:**
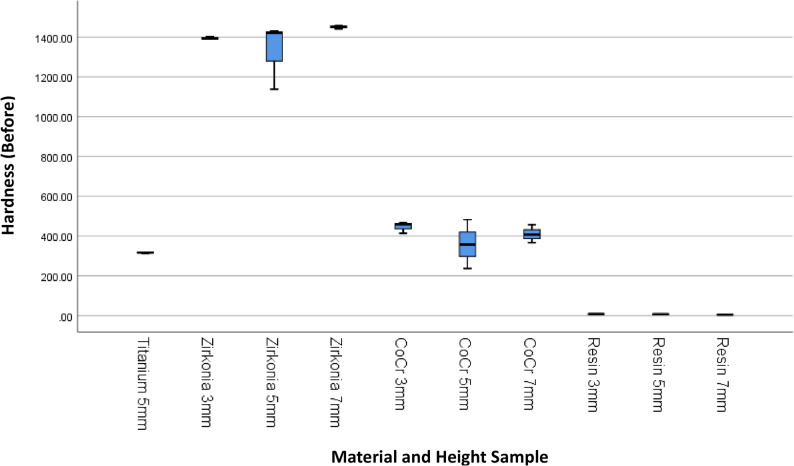
Box Plot of Hardness per Material Type and Height Before the Minimal Invasive Endodontic Pulp Chamber Preparation

**Fig. 4:**
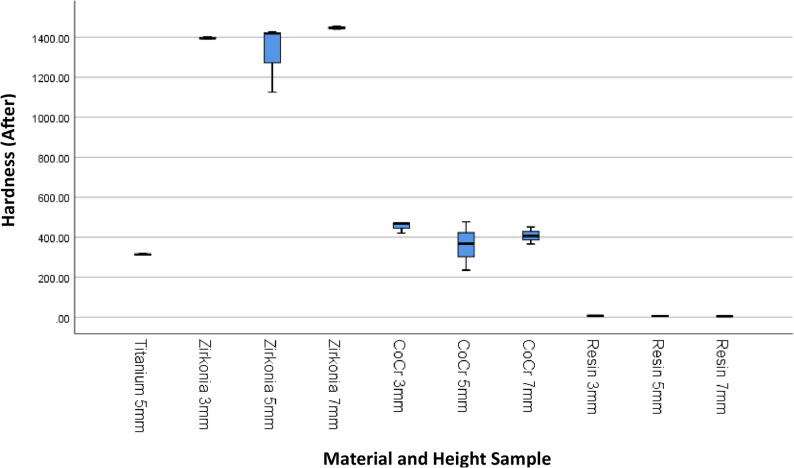
Box Plot of Hardness per Material Type and Height After the Minimal Invasive Endodontic Pulp Chamber Preparation

The results of 4 materials and 3 heights could be seen in Table 1, and Figs. 3 and 4.

Prior to pulp chamber opening, the highest average hardness was recorded in Zirconia at 3 mm (1395.16 ± 7.14), followed by Zirconia at 7 mm (1450.50 ± 9.01) and Zirconia at 5 mm (1330.66 ± 166.91). The 5 mm Titanium material exhibited a moderate hardness of 315.6 ± 2.75. The hardness of the Co–Cr material varied from 359.00 ± 123.00 (5 mm) to 446.66 ± 28.96 (3 mm). Meanwhile, the resin hardness was very low, ranging from 5.66 ± 2.08 (7 mm) to 7.66 ± 0.57 (3 mm). After pulp chamber opening, the trend of material hardness remained relatively stable. Zirconia at 3 mm continued to show the highest average hardness of 1396.33 ± 6.66, followed by Zirconia at 7 mm (1447.22 ± 6.67) and Zirconia at 5 mm (1323.33 ± 171.21). Titanium material showed only a minimal decrease to 314.55 ± 4.29. Co–Cr hardness values also remained relatively stable, with the highest average at 3 mm (453.77 ± 28.72). Resin remained the material with the lowest hardness (5.33 ± 1.52–6.67 ± 0.57). The results of the One-Way ANOVA statistical test indicated significant differences in hardness between material groups and heights before and after the invasive minimal endodontic pulp chamber opening procedure (*p* < 0.001). This result suggests that both material type and sample height significantly influence material hardness.

**Fig. 5:**
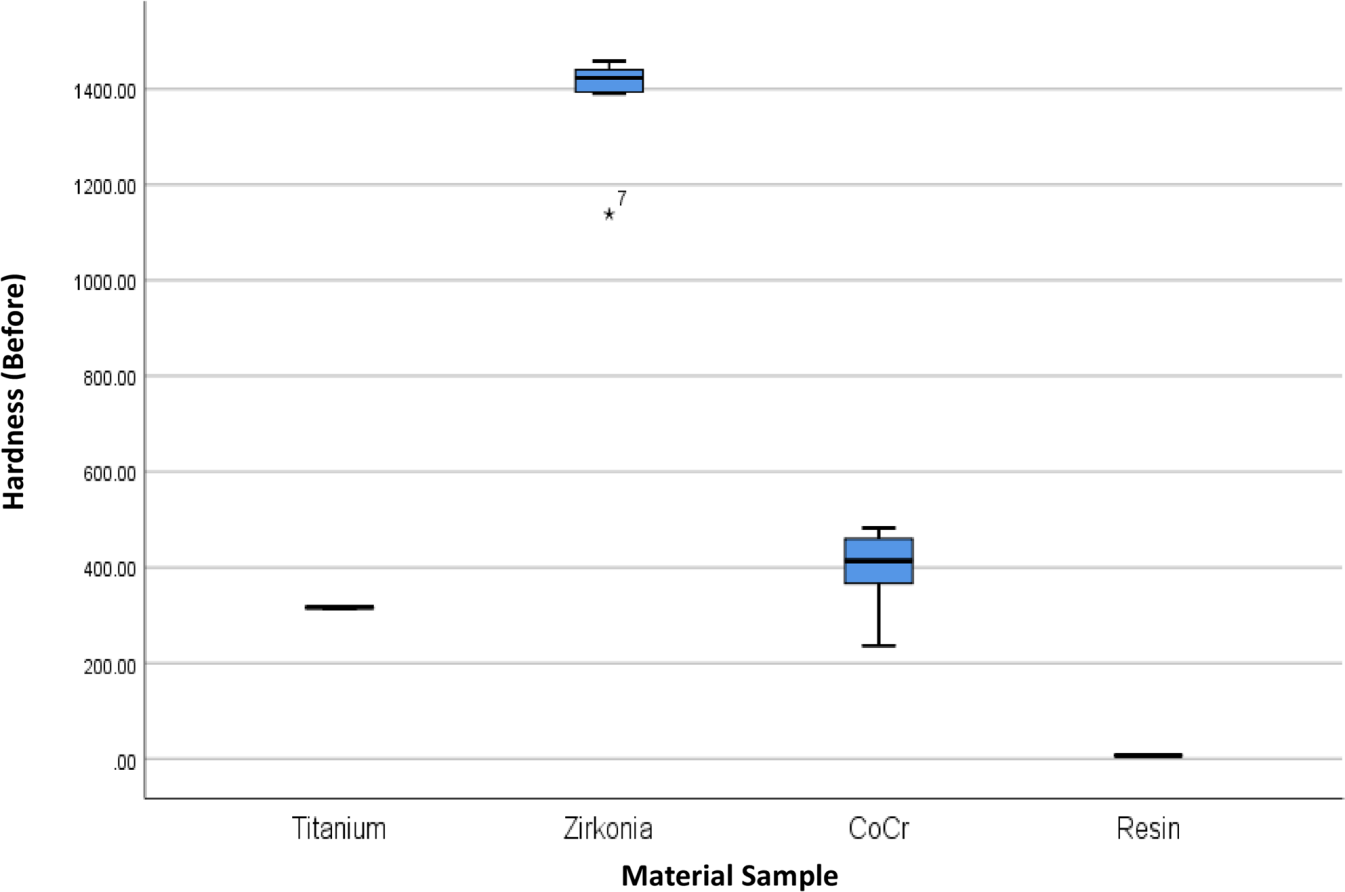
Box Plot of Hardness per Material Type Before the Minimal Invasive Endodontic Pulp Chamber Preparation

**Fig. 6:**
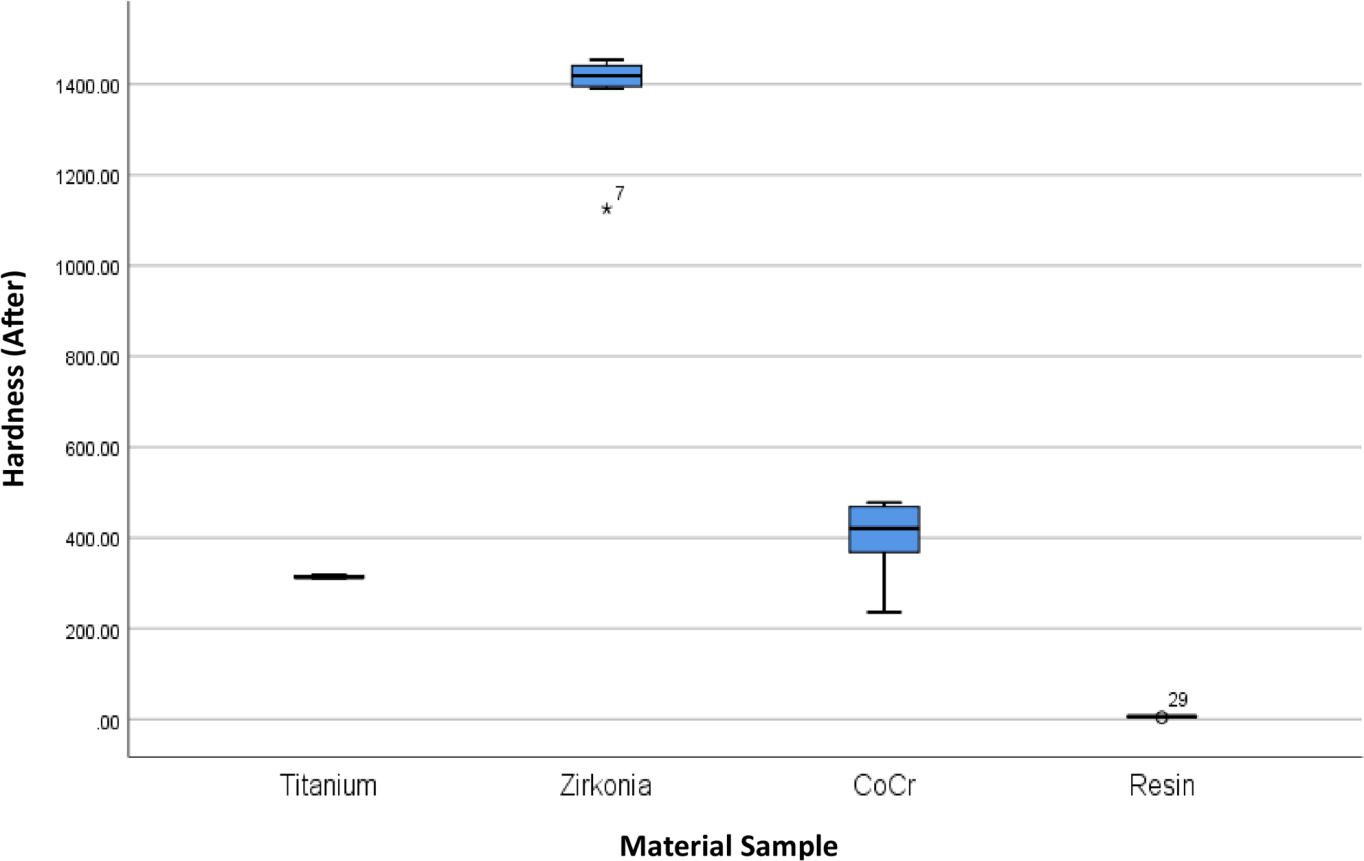
Box Plot of Hardness per Material Type After the Minimal Invasive Endodontic Pulp Chamber Preparation

This study also presents an analysis of the average hardness of each dental prosthetic material, without accounting for height variations, to provide a general overview of the influence of material on hardness before and after minimal-invasive endodontic pulp chamber opening (Table 2; Figs. 5 and 6). Before pulp chamber opening, the highest average hardness was recorded in Zirconia, at 1392.11 ± 98.46, with a median range of 1138.00–1458.00.00.00. This material exhibited significantly higher hardness compared to Titanium (315.6 ± 2.75) and Co-Cr (405.33 ± 77.20). On the other hand, Resin material had the lowest average hardness, at 6.66 ± 1.41. After pulp chamber preparation, the hardness trends among materials showed no significant changes. Zirconia remained as the highest-hardness material (1388.96 ± 101.28), followed by Co-Cr (407.48 ± 77.21) and Titanium (314.55 ± 4.29). Resin remained as the lowest-hardness material, with an average hardness of 6.11 ± 1.05. Statistical analysis using One-Way ANOVA revealed significant differences in hardness between material types, both before and after the procedure (*p* < 0.001). This result indicates that material type has a significant impact on hardness levels, and material selection is critical for mechanical stability following minimal invasive intervention.

**Table 2:** Description and comparison of hardness before and after minimal invasive endodontic pulp chamber opening by material variation

			Titanium (*n* = 3)	Zirconia (*n* = 9)	CoCr (*n* = 9)	Resin (*n* = 9)
HARDNESS	BEFORE	Mean ± SD	315.6± 2.7	1392.11 ± 98.46	405.33 ± 77.20	6.66 ± 1.41
		Median (Min-Max)	317.00 (312.50–317.50)	1,422.50 (1,138.00–1,458.00)	413.50 (236.50 −482.50)	7.00 (4.00–8.00)
		*p-value*	< 0.001			
	AFTER	Mean ± SD	314.55 ± 4.29	1388.96 ± 101.28	407.48 ± 77.21	6.11 ± 1.05
		Median (Min-Max)	313.33 (311.00–319.33)	1,419.00 (1,125.67–1,454.00)	420.66 (235.67–477.67)	6.00 (4.00–7.00)
		*p-value*	< 0.001			

*Analysis of the difference in means of normally distributed numerical data was performed using a *One-Way ANOVA* test with significance at *p* < 0.05

**Fig. 7:**
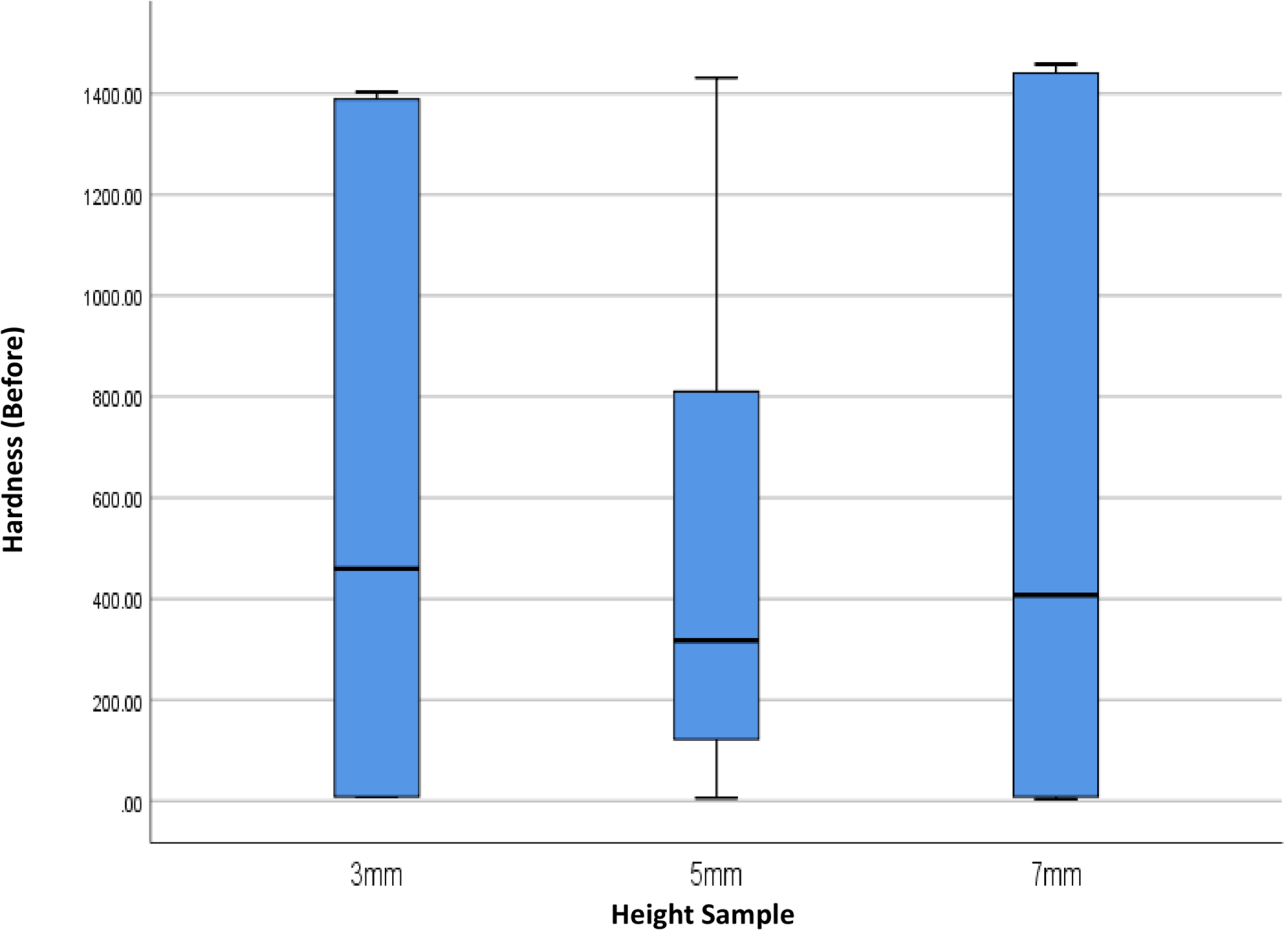
Box Plot of Hardness per Height Before the Minimal Invasive Endodontic Pulp Chamber Preparation

**Fig. 8:**
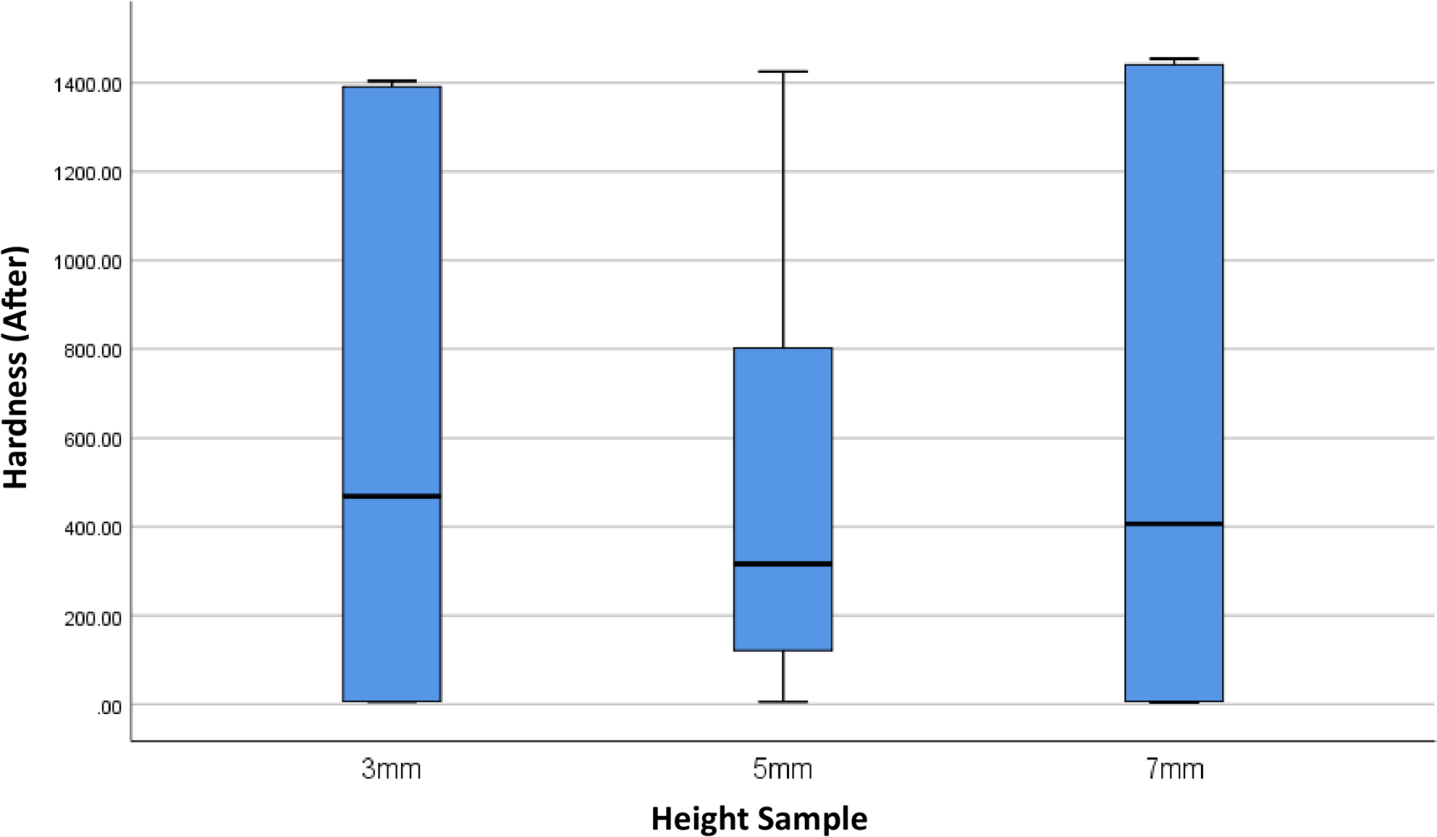
Box Plot of Hardness per Height After the Minimal Invasive Endodontic Pulp Chamber Preparation

This study also evaluated the effect of variations in the height of dental prosthetic materials (3 mm, 5 mm, and 7 mm) on hardness values, both before and after minimal invasive endodontic pulp chamber preparation (Table 3; Figs. 7 and 8). Before opening, the highest average hardness was recorded in the 7 mm height group (622.16), followed by 3 mm (616.50 ± 614.34) and 5 mm (503.00 ± 526.36). However, significant standard deviation in all groups indicates high variability in hardness values between samples. The median values at all heights were within a similar range, with the highest median at 3 mm (459.50) and the lowest at 5 mm (317.25). After the procedure, the average hardness trend remained similar, with 7 mm (620.25 ± 644.65) and 3 mm (618.92 ± 614.53) showing higher hardness compared to 5 mm (501.17 ± 523.48). The post-opening median hardness also showed a similar pattern, being higher at 3 mm (468.66) compared to 5 mm (316.33). A One-Way ANOVA statistical test indicated no significant differences in hardness between height variations, both before (*p* = 0.870) and after (*p* = 0.865) pulp chamber opening. This result means that height does not significantly affect material hardness in this study.

**Table 3:** Overview and comparison of hardness before and after minimal invasive endodontic pulp chamber opening by height variation

			3 mm (*n* = 9)	5 mm (*n* = 12)	7 mm (*n* = 9)
HARDNESS	BEFORE	Mean ± SD	616.50 ± 614.34	503.00 ± 526.36	622.16±
		Median (Min-Max)	459.50 (7.00–1403.00)	317.25 (6.00–1,431.50)	407.50 (4.00–1458.00)
		*p-value*	0.87		
	AFTER	Mean ± SD	618.92 ± 614.53	501.166 ± 523.48	620.25 ± 644.65
		Median (Min-Max)	468.66 (6.00–1403.67)	316.33 (6.00–1425.33)	407.00 (4.00–1454.00.00.00)
		*p-value*	0.86		

*Analysis of the difference in means of normally distributed numerical data was performed using a *One-Way ANOVA* test with significance at *p* < 0.05

**Fig. 9:**
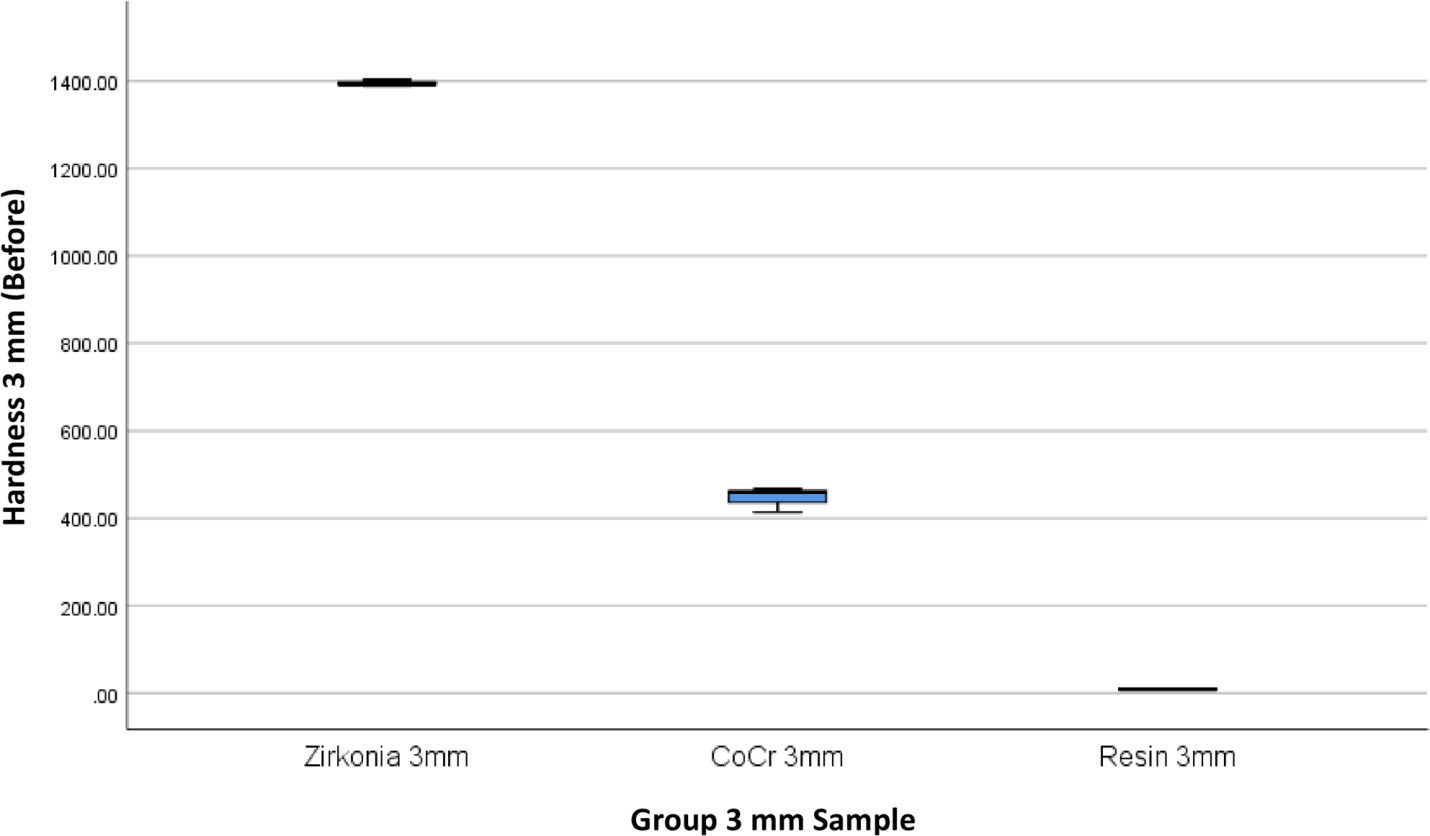
Box Plot of Hardness per Material Type and Height (3 mm) Before the Minimal Invasive Endodontic Pulp Chamber Preparation

**Fig. 10:**
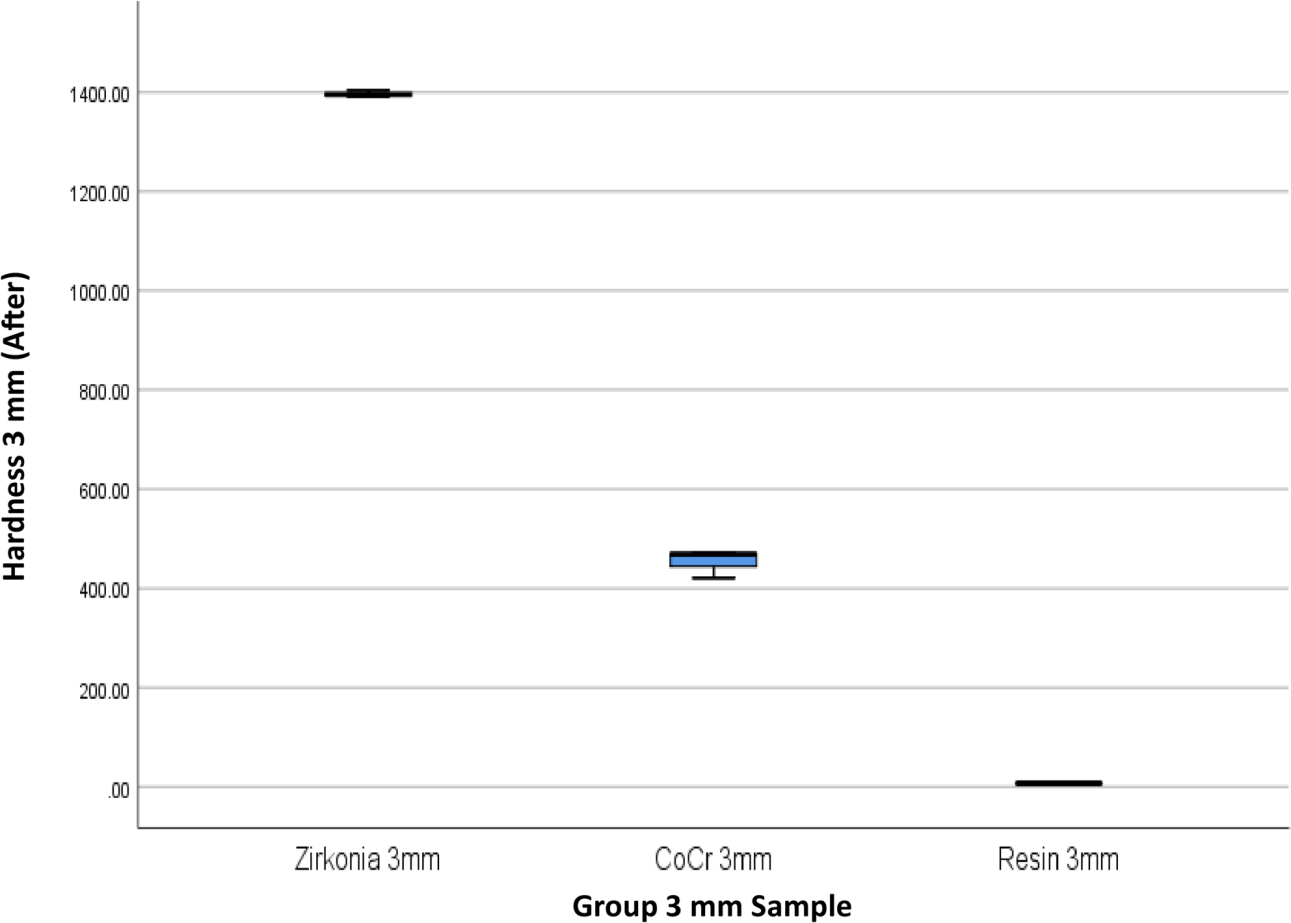
Box Plot of Hardness per Material Type and Height (3 mm) After the Minimal Invasive Endodontic Pulp Chamber Preparation

**Fig. 11:**
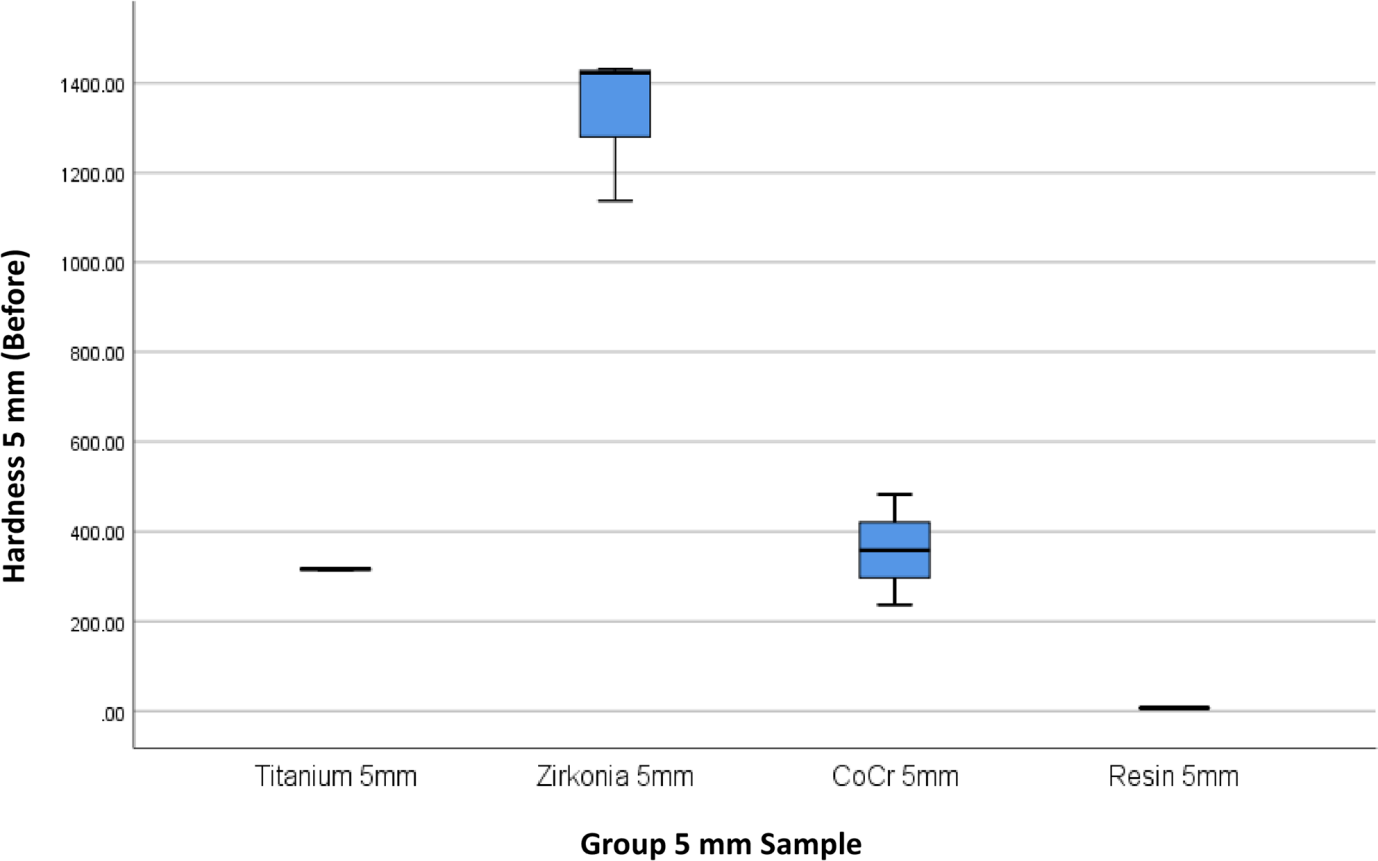
Box Plot of Hardness per Material Type and Height (5 mm) Before the Minimal Invasive Endodontic Pulp Chamber Preparation

**Fig. 12:**
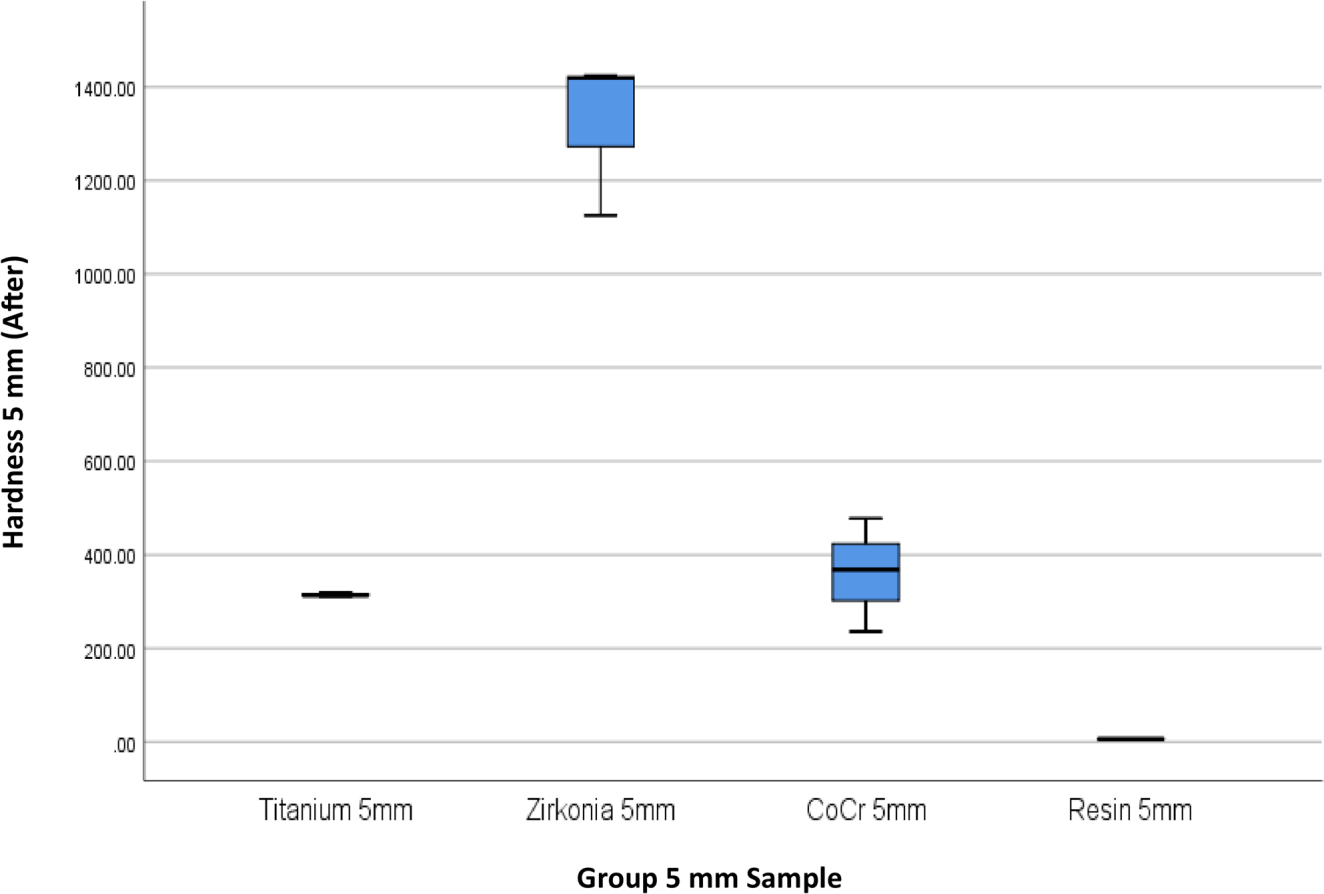
Box Plot of Hardness per Material Type and Height (5 mm) After the Minimal Invasive Endodontic Pulp Chamber Preparation

**Fig. 13:**
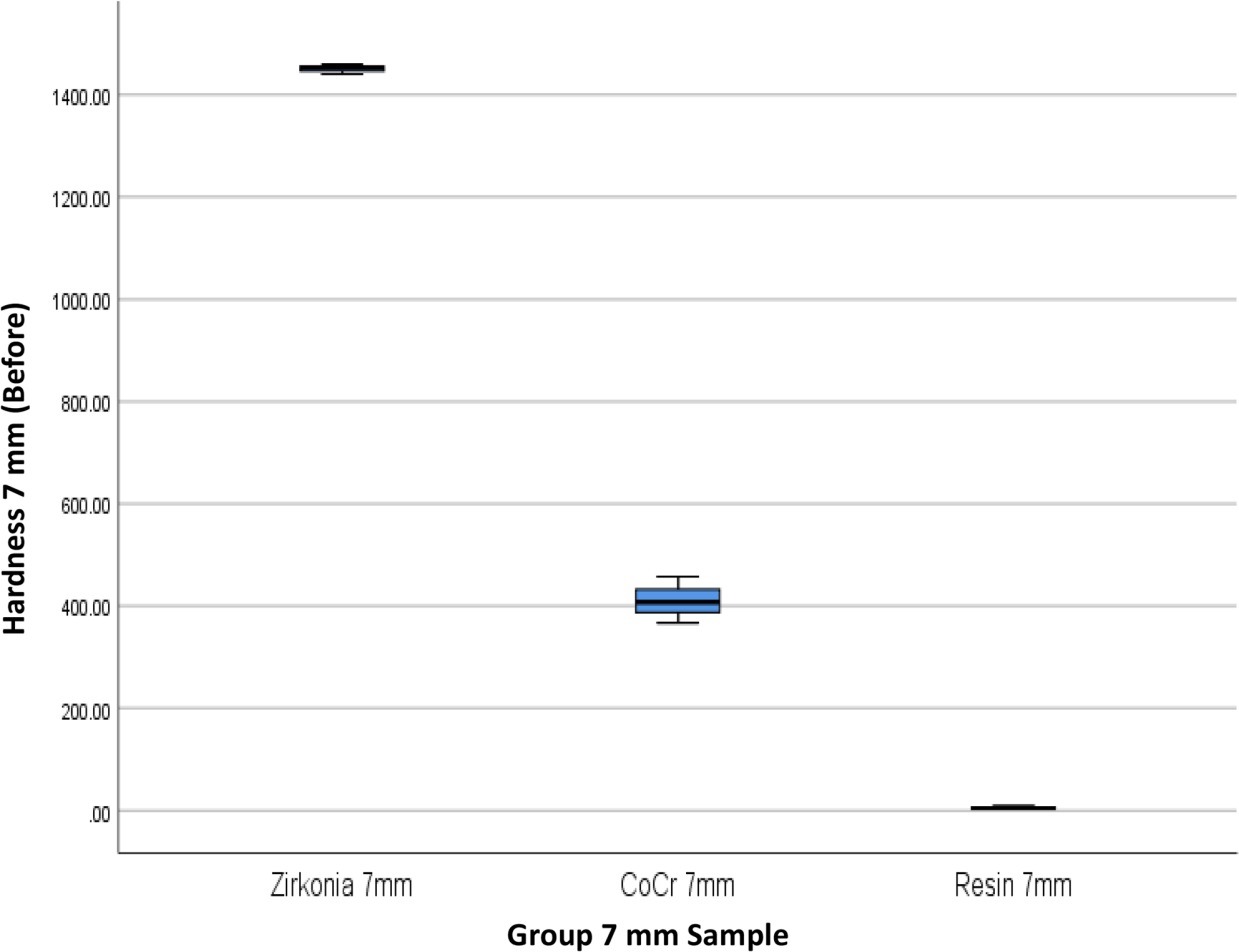
Box Plot of Hardness per Material Type and Height (7 mm) Before the Minimal Invasive Endodontic Pulp Chamber Preparation

**Fig. 14:**
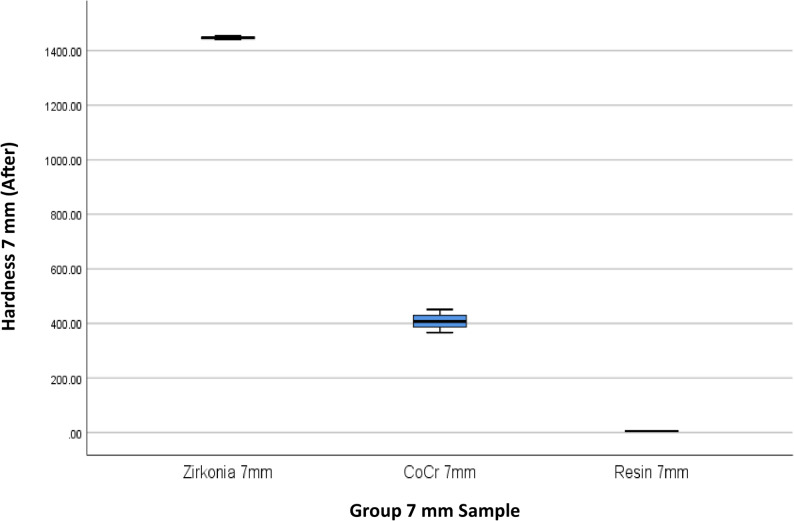
Box Plot of Hardness per Material Type and Height (7 mm) After the Minimal Invasive Endodontic Pulp Chamber Preparation

Table 4.; Figs. 9, 10, 11, 12, 13 and 14 illustrate the hardness values before and after the minimally invasive endodontic pulp chamber opening procedure on a combination of materials (Zirconia, CoCr, photopolymer resin, Titanium) and heights (3 mm, 5 mm, and 7 mm). This evaluation is important to determine which materials and structural conditions are most resistant to the minimally invasive procedure. At a 3 mm sample height, zirconia showed the highest hardness among all materials, with a Vickers hardness of 1395.16 ± 7.14 HV before the procedure and 1396.33 ± 6.66 HV after, indicating essentially no change.

**Table 4.** Overview and comparison of hardness before and after minimal invasive endodontic pulp chamber opening at each height

			3 mm			5 mm				7 mm		
			Zirconia (*n* = 3)	CoCr (*n* = 3)	Resin (*n* = 3)	Titanium (*n* = 3)	Zirconia (*n* = 3)	CoCr (*n* = 3)	Resin (*n* = 3)	Zirconia (*n* = 3)	CoCr (*n* = 3)	Resin (*n* = 3)
HARDNESS	BEFORE	Mean ± SD	1395.16± 7.14	446.6± 28.96	7.66 ± 0.57	315.6± 2.75	1,330.66± 166.91	359.00± 123.00	6.67 ± 0.57	1,450.50± 9.01	410.33 ± 45.31	5.66 ± 2.08
		Median (Min-Max)	1,393.50 (1,389.00–1,403.00)	459.50 (413.50- 467.00)	8.00 (7.00–8.00)	317.00 (312.50–317.50)	1,422.50 (1,138.00–1,431.50)	358 (236.50–482.50)	7.00 (6.00–7.00)	1,453.00 (1,440.50–1,458.00)	407.50 (366.50–457.00)	5.00 (4.00–8.00)
		*p-value*	< 0.001			< 0.001				< 0.001		
	AFTER	Mean ± SD	1396.33 ± 6.66	453.77 ± 28.72	6.67 ± 0.57	314.55 ± 4.29	1,323.33 ± 171.21	360.44 ± 121.17	6.33 ± 0.57	1,447.22 ± 6.67	408.22 ± 42.51	5.33 ± 1.52
		Median (Min-Max)	1,394.66 (1390.67- 1403.67)	468.66 (420.67- 472.00)	7.00 (6.00–7.00)	313.33 (311.00- 319.33)	1,419.00 (1,125.67–1,425.33)	368.00 (235.67–477.67)	6 (6.00–7.00)	1,447.00 (1,440.67–1,454.00)	407.00 (366.33– 451.33)	5 (4.00–7.00)
		*p-value*	< 0.001			< 0.001				< 0.001		

*Analysis of the difference in means of normally distributed numerical data was performed using a *One-Way ANOVA* test with significance at *p* < 0.05

**Fig. 15:**
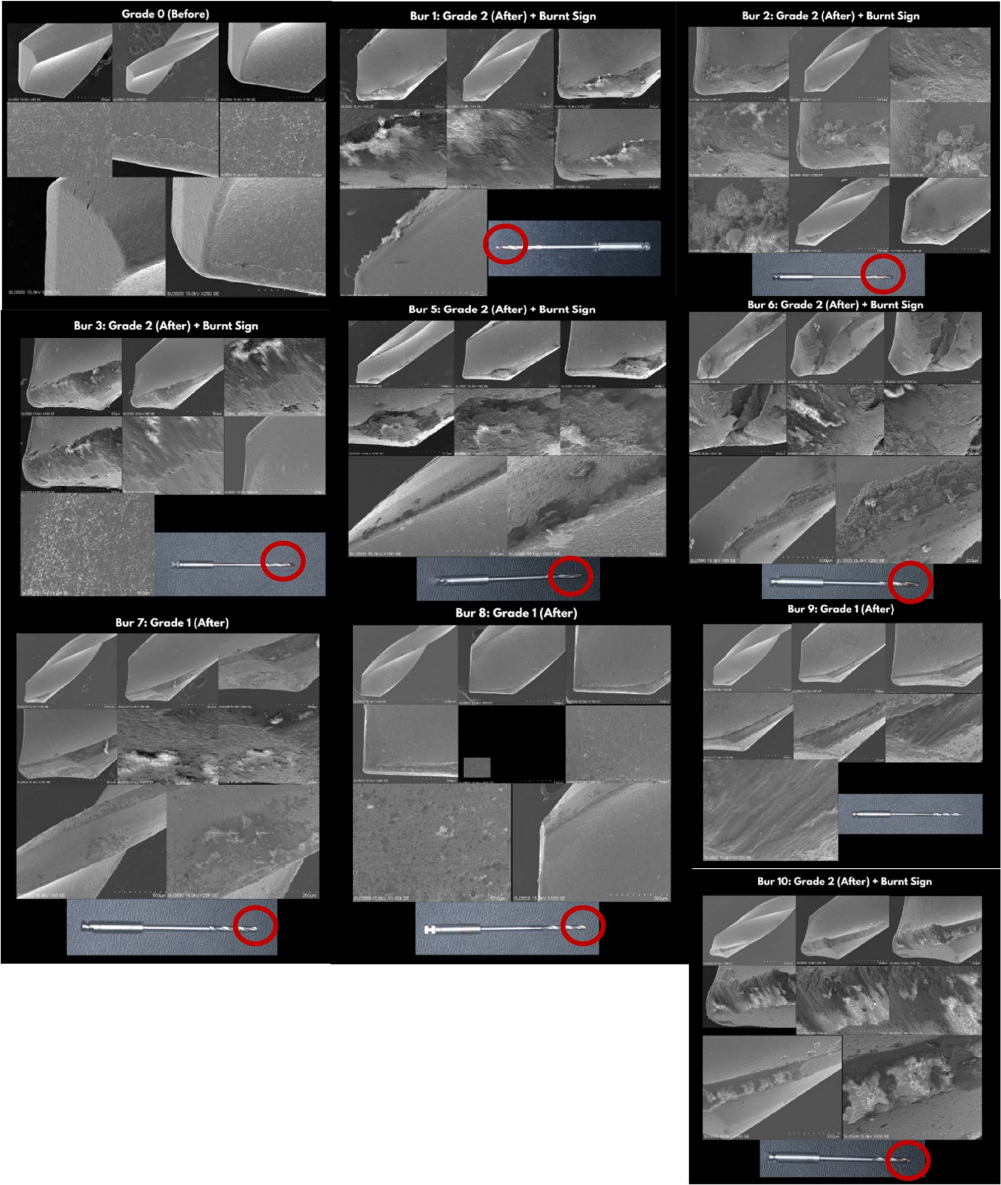
**a** Results from the Scanning Electron Microscope (SEM) on the drill before (grade 0) minimally invasive endodontic access cavity preparation, **b**. Results (Bur 1), **c**. Results (Bur 2), **d**. Results (Bur 3), **e**. Results (Bur 4), **f**. Results (Bur 5), **g**. Results (Bur 6), **h**. Results (Bur 7), **i**. Results (Bur 8), and **j**. Results (Bur 9) from the Scanning Electron Microscope (SEM) and clinical photos on the drill after (grade 2 with burnt sign (red circle)) minimally invasive endodontic access cavity preparation with a resin material endodontic sleeve guide

All examined drills showed some degree of wear (Grade ≥ 1). Six out of ten drills (70%) were scored Grade 2 (severe wear), and three (30%) were Grade 1 (moderate wear). The distribution of wear by sleeve material and height is shown in Table 5. Drills used with soft resin sleeves (3 mm, 5 mm, and 7 mm) and Zirconia (3 mm, 5 mm, and 7 mm) exhibited Grade 2 wear and burnt signs. Drills used with Titanium (5 mm) exhibited Grade 1 wear and no burnt sign. Drills used with Chrome Cobalt (3 mm, 5 mm, and 7 mm) exhibited Grades 1 and 2 wear, two burs had no burnt sign, and one bur had a burnt sign. (Table 5; Fig. 15).

**Table 5:** Wear grades of drills by sleeve material and height. (“Burnt” indicates visible thermal damage)

Sample	Material Sleeve	Wear Grade (Before)	Wear Grade (After)	Burnt
1	Resin	Grade 0	Grade 2	Yes
2	Resin	Grade 0	Grade 2	Yes
3	Resin	Grade 0	Grade 2	Yes
4	Zirkonia	Grade 0	Grade 2	Yes (Broken)
5	Zirkonia	Grade 0	Grade 2	Yes
6	Zirkonia	Grade 0	Grade 2	Yes
7	Titanium	Grade 0	Grade 1	No
8	Chrome Cobalt	Grade 0	Grade 1	No
Bur 9	Chrome Cobalt	Grade 0	Grade 1	No
Bur 10	Chrome Cobalt	Grade 0	Grade 2	Yes

## Discussion

Present study indicated that the guide sleeve material greatly affected hardness, whereas height did not. Zirconia ceramic sleeves were harder (~ 1392 HV) than CoCr (405 HV), Ti (315 HV), and polymer resin (~ 6–7 HV). These differences match the material properties: Y-TZP ceramics possess high hardness, while methacrylate polymers possess low hardness. The resin sleeve’s low hardness emphasizes its fragility, while zirconia and metals’ hardness presumably reflect bulk material qualities. Patents and designs for guide sleeves emphasize hard inner coatings (≥ 1800 HV) for abrasion resistance. Zirconia sleeves in the current study had a hardness approaching this range (tested at lesser loads), indicating their durability [7, 9]. New quantitative insights into how sleeve guide height and material affect wear resistance during guided endodontic access are presented. The toughest substance, zirconia, had essentially little wear resistance, whereas the softer resin had significant abrasion. Sleeves that were excessively short or long were unsuitable, whereas a mid-range height of 5 mm provided optimal alignment with minimal wear. Consistent hardness and low surface abrasion contributed to reduced zirconia sleeve wear. This finding indicates that smooth, high-hardness ceramics can resist bur friction without structural weakening. Zirconia’s performance in implant guides is consistent with the tribological principle that higher-hardness surfaces exhibit greater wear resistance.

Across guided-surgery literature, studies consistently show sleeves with higher hardness exhibit less wear. Ozan et al. found that zirconia sleeves lost significantly less material than CoCr sleeves during repeated drilling, [5] suggesting superior wear resistance of the zirconia. Similarly, Shruthi et al. reported that surgical guides with metal sleeves (presumably hardened steel or CoCr) showed almost no wear, whereas sleeve-free resin guides showed significantly higher drill wear (mean wear ≈ 0.0228 mm vs. 0.5036 mm, *P*< 0.05) [6]. These results align with the hardness data: the metal sleeve (hard) protected the drill, whereas the resin (soft) deformed and wore away. Chen et al. likewise supported the use of single-type guides with zirconia sleeves for optimal accuracy, [7] attributed to zirconia’s high stiffness and low wear. Notably, no prior endodontic studies report actual Vickers values for sleeves; instead, they infer hardness from wear and accuracy. Compared to previous literature, the present study provides the first direct hardness measurements (1400 HV vs. 6 HV) for typical endodontic guide materials.

Methodologically, the current study differs from others. Ozan et al. drilled implant osteotomies using *titanium drills* through prefabricated zirconia or CoCr sleeves and measured weight loss of sleeves [5]. Shruthi et al. used 3D-printed resin guides (with or without metal sleeves) and measured volumetric wear with an optical machine [6]. Chen et al. used 3D-printed surgical stents with embedded zirconia sleeves and evaluated drilling accuracy with wear roughness [7]. In contrast, the present pilot study performed *microhardness* testing (ISO HV0.2) on the sleeve inner walls before and after guided drilling, then SEM grading of drill tips. Despite methodological variations, all studies converge on a consistent finding: ceramic and metallic sleeves exhibit substantially greater hardness and wear resistance than polymer counterparts. Furthermore, Oliveira et al. (2018) reported minimal wear on CoCr sleeves and stainless-steel burs during guided surgery, consistent with the findings of the present study. Work hardening contributed to a slight increase in hardness due to localized plastic deformation. The ductility of CoCr enabled the sleeves to bear additional load without fracture. Given its high hardness and mechanical stability, CoCr remains a promising material for 3D-printed sleeves, implant systems, and prosthodontic applications [17]. 

These material differences have clear clinical implications. Higher-hardness ceramic or metal sleeves (e.g. zirconia, CoCr, Ti) should maintain their dimensions across multiple procedures, preserving guide accuracy and durability [5]. Indeed, Ozan et al. data suggest zirconia sleeves will last longer with minimal material loss, [5] and metal sleeves similarly protect the guide lumen [6]. Conversely, resin sleeves, given their very low hardness, will deform or abrade quickly, likely widening the drill channel and reducing precision over repeated use. However, the softer resin does impose less friction on the drill: resin sleeves caused less initial drill blunting (SEM Grade 1) than rigid sleeves, which caused severe (Grade 2) wear. This effect is attributed to the higher-hardness guides (zirconia/metal) holding the drill more tightly, generating greater friction and heat that accelerates drill wear.

Despite their popularity, titanium sleeves exhibited early wear. Titanium burs leave small ridges that roughen and harden the surface, as evidenced by plastic deformation at the drill surface. Repeated use of the sleeve may increase its internal diameter, thereby diminishing its accuracy in guiding the drill. Titanium sleeves are easily manufactured, but zirconia or CoCr sleeves may provide greater longevity and precision. After a single use, resin sleeves (unlined guide holes) experienced the most significant wear. Shruthi et al. found that several sleeveless resin guides shatter [6]. A single low-stress therapeutic use of a resin guide without a metal inlay may be feasible. Aging resin rapidly loses dimensional accuracy. The incorporation of carbon fibre into resin composite has improved its toughness, suggesting that reinforced polymers could serve as alternatives to metals and ceramics when those materials are unsuitable [5–8, 18]. 

In practice, clinicians must balance these trade-offs. Zirconia or CoCr sleeves offer superior wear resistance and template longevity [5], but their rigidity can cause overheating of the drill unless cooling is optimized. Resin sleeves minimize heat and drill attrition, but at the cost of sleeve wear and potential loss of guidance accuracy. Current study findings suggest selecting sleeve material based on clinical priorities: for maximal template durability and accuracy, use hard ceramic or metal sleeves, with meticulous irrigation/intermittent drilling to mitigate heat. For single-use or low-stress scenarios, softer resin sleeves may suffice, trading some precision for gentler drilling. Overall, the consensus across studies is clear: sleeve hardness correlates with durability; this directly affects guide longevity and the accuracy of guided endodontic access.

Zirconia guide sleeves are tougher and wear-resistant than polymer sleeves, which flex under tension. Caution is advised when using intermediate Ti alloys and Co–Cr materials. Ceramics or metals are preferred for reusable static guides, as polymer sleeves tend to flex or scratch, thereby reducing drilling accuracy. Sleeve fit, manufacturing costs, and other considerations matter. Sleeve integrity affects the accuracy and lifespan. Bur action may gradually wear the guide sleeve, potentially altering the drill trajectory in subsequent procedures. A widening of just 0.1–0.2 mm can reduce both depth and angular accuracy. Current research indicates that zirconia or CoCr guide sleeves help prevent drilling deviations in calcified canals. The use of multiple zirconia or CoCr sleeves enhances precision compared to titanium sleeves. Minimizing sleeve wear is essential to preventing infection during guided endodontic procedures. Residues of resin or metal within access cavities may cause tissue irritation or inflammation. Zirconia sleeves provide cleaner access with minimal material shedding. Surgeons must carefully irrigate and examine for shavings or flakes when using metal sleeves for guide constriction or heat generation. Ozan et al.’s discovery of metal particles shows that guided implant procedures require irrigation. Low-wear sleeves keep debris out of endodontic canals, where irrigation may be limited until access is achieved [5]. Geometry prevented sleeve height (3, 5, and 7 mm) from affecting hardness. Optimizing sleeve length to improve access without compromising material strength appears feasible. Even when structurally sound, extended sleeves may limit access in posterior regions. The homogeneous drilling procedure used in the present study may explain why drilling pressure remained constant regardless of sleeve length. Hardness remained consistent across different sleeve heights.

In this SEM study, 70% of endodontic access drills showed significant wear after guided use. In severe cases, resin, zirconia, and CoCr sleeves classified as Grade 2 exhibited signs of thermal damage. Grade 1 wear was observed in drills used with CoCr and titanium sleeves. To avoid overheating, Prabhuji et al. advised irrigation cooling. Sleeve height influenced wear. Short-sleeved (little guidance) groups kept drills at Grade 1 owing to minimal surface contact. Medium/long sleeves increased friction by stabilizing the drill over longer lengths. Prabhuji et al. found that longer sleeves may block irrigation, preventing coolant from reaching the bur [14]. Yang et al. found that sleeves above 5 mm may prevent coolant from reaching the bur, leading to overheating. All drills in the current study exhibiting severe thermal damage corroborated these findings [14, 19]. In conclusion, although higher sleeves provide better stability, they may also increase heat buildup and tool wear, particularly when made from high-hardness materials.

The drill and inner sleeve became heated due to friction. Contact friction rapidly generates heat, potentially causing thermal damage to the bur or sleeve [20, 21]. Frictional heat may cause burning of the sleeve and bur when drilling duration is prolonged and irrigation is insufficient. Therefore, appropriate irrigation planning is important during drilling. Previous research indicates that incorporating a cooling system with root canal irrigation during guided endodontic access preparation is important to minimize drill damage and overheating. The absence of a cooling device during root canal irrigation caused severe damage to all drills in the present study. Zirconia sleeves exhibited minimal wear despite continuous use, as reported by Ozan et al. [5–7].

The present investigation found that resin sleeves can protect drills in specific applications, yet drill wear and overheating remain major clinical concerns. Poorly sharpened drills may fracture quickly, and excessive heat can damage surrounding tissues. In this ex vivo study, a single drilling cycle per drill still resulted in 60% hole damage, underscoring the need for regular drill replacement when using hard-metal sleeves. Frictional heat, coolant delivery, and heat dissipation are the main thermal factors influenced by the guide sleeve. Metal sleeves often increase friction and trap debris, reducing coolant flow and delaying heat discharge, while sleeveless or shorter guides generate less heat but may lower accuracy [5–7, 14, 19]. Heat rises sharply during drilling in calcified dentin, which lacks blood flow to disperse it. To minimize temperature elevation, clinicians should apply intermittent “pecking” drilling, adequate irrigation, and slower rotational speeds (800–1000 rpm). Pre-access cavity preparation also reduces temperature by up to 50%. Using sharp, single-use drills matched to sleeve diameter, combined with optimized 3D guide planning and intraoperative verification, can further control frictional heat. Overall, careful control of speed, cooling, and guide design helps prevent thermal injury and enhances the safety of guided endodontic access procedures [13, 22]. 

Limitations of this in vitro study included the use of extracted human tooth analogy (gypsum blocks) and controlled laboratory conditions. The effect of anatomical variation, intraoral temperature, and multiple usage cycles was not tested. Only one drilling cycle per sleeve was performed; repeated use (and sterilization) could yield different results. Hardness was measured at a single depth; microstructural effects at the sleeve surface (such as microfractures) were not assessed. Future work should examine sleeve wear (mass loss) under multiple uses and temperature changes, and its impact on drilling accuracy. Subsequent studies could compare hardness after repeated drilling cycles to simulate clinical reuse, include dynamic (rotating handpiece) guidance systems, and correlate hardness changes with guide deviation in actual teeth. Investigations into coating technologies or surface treatments for 3D-printed sleeves could be valuable, given the low hardness of the resin. In vivo studies might evaluate wear particles generated from sleeves and potential biocompatibility issues.

The limitations of this SEM study include a small sample size of 10 drills and the use of qualitative scoring. Only a single drill was utilized per group, thus precluding the assessment of individual drill variation. Actual cutting efficiency and temperature rise were not measured. The selected sleeve heights served illustrative purposes and did not encompass all clinical guide designs. Future research should quantify wear volumes, for instance, through micro-CT, and assess intraroot temperatures across different irrigation strategies. It would be beneficial to compare repeated drilling cycles with new drill materials, such as ceramic drills.

Future research should focus on optimizing guided endodontic systems by evaluating drill wear under repeated use and sterilization to simulate clinical reuse, and by developing advanced sleeve materials such as carbon-reinforced polymers, PEEK, or coated alloys that balance durability with low friction. Biomechanical studies should assess how sleeve height, rigidity, and fit tolerance influence drill trajectory, heat generation, and irrigation efficiency. In vitro thermal analyses and in vivo trials are needed to quantify temperature rise, wear debris, and biocompatibility effects. Furthermore, exploration of sleeveless or dynamic navigation systems could reveal safer, more precise alternatives that minimize thermal injury while maintaining accuracy.

## Conclusion

Sleeve material significantly influenced hardness and drill wear, while sleeve height showed no effect. Zirconia possesses the highest hardness, followed by CoCr, titanium, and resin. Although hardness remained stable after drilling, sleeves with higher hardness tended to increase drill wear and heat generation. Proper sleeve selection and adequate irrigation are essential to enhance safety and accuracy in guided endodontics.

## Data Availability

The datasets used and analyzed during the current study are available from the corresponding author upon reasonable request.

## Abbreviations

ANOVA: Analysis of variance
CBCT: Cone-beam computed tomography
CoCr: Cobalt-chromium alloy
SD: Standard deviation
Ti–6Al–4V: Titanium-6%Al-4%V alloy
VHN: Vickers hardness number
Y-TZP: Yttria-stabilized tetragonal zirconia polycrystal
